# Minimally invasive extracorporeal circulation in end-stage coronary artery disease patients undergoing myocardial revascularization

**DOI:** 10.1186/s13019-021-01735-0

**Published:** 2021-12-27

**Authors:** Ignazio Condello, Giuseppe Santarpino, Francesco Bartolomucci, Giovanni Valenti, Nicola Di Bari, Marco Moscarelli, Vincenza Vitobello, Vera Triggiani, Mario Gaudino, Flavio Rimmaudo, Giuseppe Speziale, Giuseppe Nasso

**Affiliations:** 1 Department of Cardiac Surgery, Perfusion Service, GVM Care and Research, Anthea Hospital, Via Camillo Rosalba 35/37, 70124 Bari, Italy; 2 Department of Cardiac Surgery, Paracelsus Medical University, Nuremberg, Germany; 3 Cardiac Surgery Unit, Department of Experimental and Clinical Medicine, University “Magna Graecia” of Catanzaro, Catanzaro, Italy; 4 ASL BAT, Andria, Italy; 5 Division of Cardiac Surgery, Dipartimento di Emergenza e Trapianti di Organo (D.E.T.O.), University of Bari, Bari, Italy; 6 Department of Cardiothoracic Surgery, Weill Cornell Medicine, New York, NY USA

**Keywords:** Minimally invasive extracorporeal circulation, MiECC, End-stage coronary artery disease, Coronary artery bypass grafting

## Abstract

**Background:**

Patients with coronary artery disease and concomitant heart failure (left ventricular ejection fraction < 35%) requiring myocardial revascularization are at risk of poor long-term prognosis and higher mortality. The benefits of minimally invasive extracorporeal circulation (MiECC), particularly in end-stage coronary artery disease patients undergoing myocardial revascularization, have not been completely described.

**Materials and methods:**

In this single-centre control study, 60 end-stage coronary artery disease patients undergoing isolated coronary artery bypass grafting (CABG) were included. Patients were divided into two groups of 30 patients each undergoing CABG using MiECC or conventional extracorporeal circulation (cECC).

**Results:**

In the MiECC group, oxygen delivery index (DO_2i_) was 305 mL/min/m^2^ in relation to indexed oxygen extraction ratio (O_2_ER_i_) 21.5%, whereas in the cECC group DO_2i_ was 288 mL/min/m^2^ in relation to O_2_ER_i_ 25.6% (*p* = 0.037). Lactate levels > 3 mmol/L were reported in 7 MiECC patients vs 20 cECC patients (*p* = 0.038), with blood glucose peak. Mean nadir hemoglobin values during cardiopulmonary bypass (CPB) were 9.7 g/dL in the MiECC group vs 7.8 g/dL in the cECC group (*p* = 0.044). Cardiac index during CPB was 2.4 L/min/m^2^ in both groups. Red blood cell units administered were 8 vs 21 units in the MiECC vs cECC group (*p* = 0.022). A glycemic peak was recorded in 7 patients of the MiECC group and in 20 patients of the cECC group (*p* = 0.037).

**Conclusion:**

In end-stage coronary artery disease, the MiECC technique was associated with a higher DO_2i_ compared to cECC. MiECC patients showed a significant reduction in red blood cell unit administration and peak intraoperative lactate levels, which correlated with better postoperative outcome.

## Introduction

Patients with coronary artery disease and concomitant heart failure (left ventricular ejection fraction [LVEF] < 35%) requiring myocardial revascularization are at risk of poor long-term prognosis and higher mortality [1]. In this population, the appropriate selection of perioperative techniques and strategies is crucial for the prevention of acute kidney injury (AKI) that frequently occurs after cardiopulmonary bypass (CPB).

The management and monitoring of metabolic parameters during extracorporeal circulation (ECC) has gained widespread adoption over the years, particularly in relation to the target values of oxygen delivery (DO_2_) > 262 mL/min/m^2^, carbon dioxide production > 5.3, indexed oxygen extraction ratio (O_2_ER_i_) < 25% [2–4], with average blood pressure values during CPB of 50–70 mmHg. This has made it possible to reduce the incidence of postoperative AKI and to improve the management of aerobic vs anaerobic metabolism during cardiac surgery procedures. At the same time, minimally invasive ECC (MiECC) technologies have been developed and introduced into clinical practice [5].

The aim of this study was to compare MiECC vs conventional ECC (cECC) in patients with end-stage coronary artery disease undergoing myocardial revascularization.

## Material and methods

### Population and study design

Between February 2020 and May 2021, 60 patients aged > 75–83 years with a mean EuroSCORE II of 9.1–9.5% and LVEF < 35% underwent myocardial revascularization at our institution. A retrospective comparison was carried out in terms of maximum DO_2_ and O_2_ER_i_ < 25% for standard cardiac index (CI) of 2.4 (l/min/m^2^). Patients were divided into two groups: 30 patients underwent coronary artery bypass grafting (CABG) using MiECC and 30 patients underwent CABG using cECC (Table 1). Metabolic management through blood gas analysis integrated with the use of a metabolic parameter monitoring system during CPB was adopted in both groups.

**Table 1:** Preoperative characteristics

	MiECC (n = 30)	cECC (n = 30)	*p* value
Age (years), mean	78.7	79.3	0.93
Male sex, *n* (%)	22 (73)	21 (70)	0.91
Body surface area (m^2^), mean	1.79	1.78	0.99
Left ventricular ejection fraction (%), mean	28	29	0.89
EuroSCORE II (%)	9.1	9.5	0.92
Pre-CPB Hct (%), mean ± SD	32.4 ± 1.2	32.6 ± 1.9	0.92
Pre-CPB Hb (g/dL), mean ± SD	11.7 ± 1.1	11.8 ± 1.2	0.94
Chronic obstructive pulmonary disease, *n*	6	5	0.96
Creatinine (mg/dL), mean ± SD	1.14 ± 0.2	1.13 ± 0.5	0.98
Obstructive coronary artery disease	30	30	1
Peripheral arterial disease	2	3	0.98

cECC, conventional extracorporeal circulation; MiECC, minimally invasive extracorporeal circulation; CPB, cardiopulmonary bypass; Hb, hemoglobin; Hct, hematocrit; SD, standard deviation

The study protocol was approved by the local ethics committee (session June 2021) and all patients provided written informed consent to data treatment.

### Data collection

Patients were selected according to the following criteria:

- Patients with coronary artery disease and concomitant heart failure (LVEF < 35%) requiring myocardial revascularization, in whom complete CPB and cardioplegic arrest had to be foreseen with an expected CPB duration > 90 min.
- Patients were excluded if they presented abnormal plasma lactate levels (> 2 mmol/L) before entering CPB, liver failure, obesity, uncompensated diabetes, autoimmune disease, active infection, any immunosuppressive therapy, or coagulation disorder. Patients undergoing combined surgery (e.g. aortic valve replacement + CABG, about 300 patients during the study period) or surgery with circulatory arrest or having preoperative hematocrit (Hct) < 27% were also excluded.

All CABG procedures (n = 100) were analyzed for this study. Preoperative data included patient demographics, baseline serum creatinine, LVEF, comorbidities (chronic obstructive pulmonary disease, previous cerebrovascular accident), baseline hemoglobin (Hb), EuroSCORE II and New York Heart Association functional class [2].

Perioperative data included type of operation, CPB duration, nadir body temperature during CPB, nadir Hct and Hb values (measured at the start of CPB and every 20 min thereafter), nadir DO_2_ index (DO_2i_), nadir DO_2i_/O_2_ER_i_ ratio during CPB, nadir CI, nadir CI/mixed venous oxygen saturation (SvO_2_), peak serum lactate and glucose during CPB and perioperative administration of red blood cell units.

Postoperative data included peak serum creatinine, mechanical ventilation time and days spent in the intensive care unit (ICU).

The primary endpoints were: maximum DO_2i_ in relation to O_2_ER_i_ during CPB compared between groups in terms of intraoperative lactate and glycemia trends. Secondary endpoints were total red blood cell units transfused, peak postoperative serum creatinine [6–8], mechanical ventilation time, and length of ICU stay.

### Anesthetics and surgical procedures

Patients were monitored with five-lead electrocardiography, a left radial artery catheter, capnography, pulse oximetry, and rectal/urine bladder temperature sensors. Transesophageal echocardiography was performed in all patients. Anticoagulant therapy consisted of heparin sodium before CPB at 300 IU/kg to achieve an activated clotting time of > 480 s (ACT PLUS Medtronic, Minneapolis, MN, USA); for heparin neutralization, 0.5–0.75 mg protamine was given for every 100 heparin units. Anesthesia was induced with intravenous sufentanil (0.5–1 μg/kg) and midazolam (0.08–0.2 mg/kg), and tracheal intubation was facilitated with intravenous rocuronium (0.6–1 mg/kg). Anesthesia was maintained with propofol (2–5 mg/kg) and sufentanil (0.5–2.0 μg/kg), and bispectral index values (BIS XP, Aspect Medical System, Newton, MA, USA) were used for depth of anesthesia monitoring. The dosage of propofol was titrated to maintain bispectral index values between 40 and 60. Aortic valve replacement and CABG procedures were performed in median sternotomy with central cannulation, and surgical procedures were performed as routine by two surgeons. Concentrated red blood cells were transfused whenever Hb concentrations fell below 6 g/dL during surgery or below 8 g/dL during ICU stay. The goal of hemoconcentration was to eliminate the excess of crystalloid administration.

### Cardiopulmonary bypass setting

#### MiECC group

**Fig. 1:**
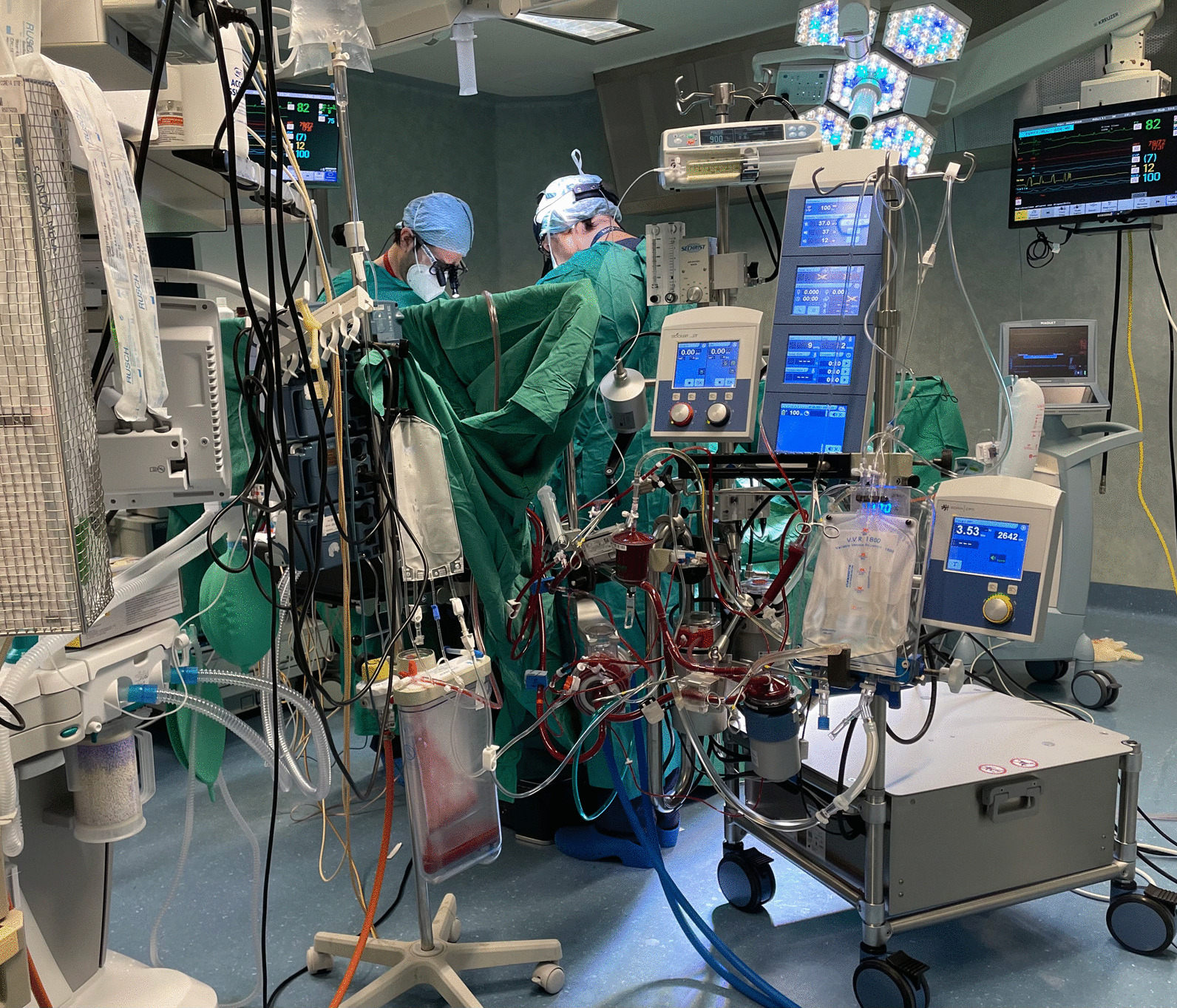
Minimally invasive extracorporeal circulation (MiECC) during myocardial revascularization in end-stage coronary artery disease patients

A closed MiECC type III circuit was employed using Stöckert S5 heart-lung machine (LivaNova, London, UK) [5], whose design presents the characteristics of a volume management circuit (MiECTiS classification). A shunted venous soft-shell reservoir (Closed, Eurosets, Medolla, Italy) was used, aortic root and pulmonary artery venous suction drainage was managed in sequence. The components (Biopassive Coating Phisio, LivaNova, London, UK) (Fig. 1) were as follows: venous-arterial line diameter (3/8), venous bubble-trap (Sherlock, Eurosets), a centrifugal pump (Biomedicus BPX80, Medtronic, Eden Prairie, MN, USA), and a polypropylene fiber oxygenator (Alone, Eurosets). A bubble detection system was used to remove the air from the bubble trap and the circuit (Stockert, LivaNova). Circuit filling volume 500 mL crystalloid solution. 300 IU/kg of sodium heparin were administered, the activated clotting time prior to CPB was 501 s, the cannulas were connected to the air-free circuit, and the bypass with a closed system was set up, the reference value of management of venous drainage was central venous pressure, maintained around 5 mmHg using urapidil as a vasodilator for higher values, or upon request of drainage by the surgeon, the Trendelenburg position was used for lower values [4, 5, 9, 10]. All patients were treated with mild hypothermic CPB (34–36 °C). For the administration of myocardial protection, a closed circuit for cardioplegia with heat exchanger, with an infusion syringe pump and Saint Thomas solution with procaine were used and repeated every 30 min. The Landing monitoring system (Eurosets, Medolla, Italy) was used for DO_2_ management during CPB. In both groups, blood gas analyses were performed using alpha-stat management with a blood gas analyzer (GEM Premier 3000 IQM, Instrumentation Laboratory, Werfen Group IVD company, Munchen, Germany) set to measure at 37 °C [11]. On the basis of arterial blood data, we assessed the lowest Hct (percentage) on CPB; every 20 min, an arterial blood gas analysis, including blood glucose (mg/dL) and lactate (mmol/L) determination, was obtained. An Hb value < 6 g/dL during CPB was considered the trigger point for red blood cell transfusion. All patients received tranexamic acid according to routine protocol. Mean arterial pressure during CPB procedures was managed for values between 55 and 70 mmHg.

#### cECC group

Open circuits with roller pumps (Admiral, Remo-well Eurosets, Medolla, Italy; Inspire 6 F, LivaNova, London, UK) were used for CPB. Pericardial blood was collected separately and could be processed or reinjected, if needed. The hard shell and softshell reservoir, oxygenating module and circuits were treated with phosphorylcholine (Agile Eurosets, Medolla, Italy; Phisio, LivaNova, London, UK). All patients were treated with mild hypothermic CPB (34–36 °C); a volume of 1250 mL crystalloid Ringer acetate solution was used for priming. The surgical procedures selected for this study do not justify the use of moderate hypothermia by falling below 34 °C. For this reason, in the event of an initial increase in anaerobic metabolism, the first compensation approach was not to lower the temperature but possibly liquids or red blood cells were integrated.

The hardware consisted of a Stöckert S5 heart-lung machine and a Stöckert Heater Cooler System 3 T (LivaNova, London, UK) and the same cannulae were employed in both groups. For the administration of myocardial protection, a closed circuit for cardioplegia with heat exchanger, with an infusion syringe pump in sequence and Saint Thomas solution with procaine were used and repeated every 30 min. The Landing monitoring system (Eurosets, Medolla, Italy) was used for DO_2_ management during CPB. In both groups, blood gas analyses were performed using alpha-stat management with a blood gas analyzer (GEM Premier 3000 IQM, Instrumentation Laboratory, Werfen Group IVD company, Munchen, Germany) set to measure at 37 °C [11]. On the basis of arterial blood data, we assessed the lowest Hct (percentage) on CPB; every 20 min, an arterial blood gas analysis, including blood glucose (mg/dL) and lactate (mmol/L) determination, was obtained. An Hb value < 6 g/dL during CPB was considered the trigger point for red blood cell transfusion. All patients received tranexamic acid according to routine protocol. As in the MiECC group, mean arterial pressure during CPB procedures was managed for values between 55 and 70 mmHg.

### Statistics

Statistical analysis was made according to Hickey’s criteria. Continuous variables are presented as mean and standard deviation. As descriptive statistics, our results are not reported as inferential statistics. We used a routine statistical method, i.e. Student’s t-test, which does not require extensive details.

## Results

Demographic, preoperative and operative details of the patient population are shown in Tables 1 and 2. There were no difference between groups in preoperative characteristics; all patients had LVEF < 35% and underwent isolated CABG with prior risk assessment (EuroSCORE II 9.1–9.5%).

**Table 2:** Operative data

	MiECC (n = 30)	cECC (n = 30)	*p* value
CPB time (min), mean ± SD	115 ± 9.2	110 ± 6.17	0.93
Aortic cross-clamp time (min), mean ± SD	71 ± 4	69 ± 6	0.83
Nadir temperature during CPB (°C), mean ± SD	34.9 ± 1.1	34.7 ± 2.1	0.75
Nadir Hb value during CPB (mg/dL), mean ± SD	9.7 ± 1.5	7.8 ± 1.2	0.044
Nadir Hct during CPB (%), mean ± SD	29.8 ± 0.3	25.1 ± 2.1	0.043
Nadir Hb after CPB (mg/dL), mean ± SD	9.4 ± 0.1	7.2 ± 0.8	0.044
Nadir Hct after CPB (%), mean ± SD	29.2 ± 0.1	24.3 ± 0.9	0.045
Nadir DO_2i_ during CPB (mL/min/m^2^), mean ± SD	305 ± 9	288 ± 6	0.037
O_2_ER_i_ during CPB (%), mean ± SD	20 ± 1	25 ± 3	0.0029
Nadir CI during CPB (L/min/m^2^), mean ± SD	2.4 ± 0.2	2.4 ± 0.1	0.94
Nadir SvO_2_ (%)	81 ± 2	75 ± 5	0.038
Crystalloid solution (mL)	328 ± 41	727 ± 57	0.039
Red blood cells (units)	8	21	0.021
Red blood cells during CPB (units)	3	10	0.023
Red blood cells during ICU stay (units)	5	11	0.024

cECC, conventional extracorporeal circulation; MiECC, minimally invasive extracorporeal circulation; CI, cardiac index; CPB, cardiopulmonary bypass; DO_2i_, indexed oxygen delivery; Hb, hemoglobin; Hct, hematocrit; ICU, intensive care unit; O_2_ER_i_, indexed oxygen extraction ratio; SD, standard deviation; SvO_2_, mixed venous oxygen saturation

In the MiECC group, oxygen delivery index (DO_2i_) was 305 mL/min/m^2^ in relation to indexed oxygen extraction ratio (O_2_ER_i_) 21.5%, whereas in the cECC group DO_2i_ was 288 mL/min/m^2^ in relation to O_2_ER_i_ 25.6% (*p* = 0.037). Lactate levels > 3 mmol/L were reported in 7 MiECC patients vs 20 cECC patients (*p* = 0.038), with blood glucose peak (Table 3). Mean nadir Hb values during CPB were 9.7 g/dL in the MiECC group vs 7.8 g/dL in the cECC group (*p* = 0.044). CI during CPB was 2.4 L/min/m^2^ in both groups. As for liquid administration, including anesthesia infusions, 727 mL and 328 mL of crystalloid solution were given to MiECC and cECC patients, respectively (*p* = 0.039) (Table 2). Red blood cell units administered were 8 vs 21 units in the MiECC vs cECC group (*p* = 0.022). A glycemic peak was recorded in 7 patients of the MiECC group and in 20 patients of the cECC group (*p* = 0.037). Patients with hyperlactatemia during CPB showed a significant increase in serum creatinine [7], higher rate of prolonged mechanical ventilation and longer ICU stay (Table 4). No patient underwent ultrafiltration during CPB.

**Table 3:** Peak blood lactate and DO_2i_ in relation to O_2_ER_i_ during cardiopulmonary bypass in the MiECC and cECC groups

	No hyperlactatemia or hyperglycemia	Hyperlactatemia and hyperglycemia
**MiECC**		
No. patients	23	7
Peak blood lactate (mmol/L)	1.08 ± 0.19	1.93 ± 0.25
Mean DO_2i_ (mL/min/m^2^)	304 ± 21	275 ± 19
Mean O_2_ER_i_ (%)	20 ± 3	38 ± 4
Blood glucose (mg/dL)	129 ± 9	205 ± 11
**cECC**		
No. patients	10	20
Peak blood lactate (mmol/L)	1.28 ± 0.45	3.91 ± 1.21
Highest DO_2i_ (mL/min/m^2^)	289 ± 11	265 ± 19
Highest O_2_ER_i_ (%)	25 ± 3	33 ± 4
Blood glucose (mg/dL)	149 ± 3	230 ± 11

Values are given as mean ± standard deviation. cECC, conventional extracorporeal circulation; DO_2i_, indexed oxygen delivery; MiECC minimally invasive extracorporeal circulation; O_2_ER_i_, indexed oxygen extraction ratio

**Table 4:** Hyperlactatemia during cardiopulmonary bypass and postoperative outcome

	MiECC (n = 30)		cECC (n = 30)	
	No HL (n = 23)	HL (n = 7)	No HL (n = 10)	HL (n = 20)
Peak serum creatinine (mg/dL)	1.1 ± 0.1	1.4 ± 0.5	1.19 ± 1.1	1.7 ± 1.5
MV time (h)	19.6 ± 45	55 ± 31	22.6 ± 55	52 ± 49
ICU stay (days)	1.2 ± 2.1	5.2 ± 4.9	1.5 ± 2.1	6.1 ± 2.9

Values are given as mean ± standard deviation. cECC, conventional extracorporeal circulation; MiECC minimally invasive extracorporeal circulation; HL, hyperlactatemia; ICU, intensive care unit; MV, mechanical ventilation

## Discussion

This retrospective study aimed at comparing two different CPB techniques (i.e. MiECC type III vs cECC) in end-stage coronary artery disease patients undergoing myocardial revascularization in terms of DO_2i_ values in relation to O_2_ER_i_ with the same target CI, and of incidence of peak lactate and correlation with postoperative outcome. More specifically, the type of ECC technique can influence intraoperative DO_2i_ with blood product use but hemodilution being equal. In other words, mean DO_2_i was higher in the MiECC group compared to the cECC group with higher Hb and Hct, though with less transfusions administered since the flow rate of the two circuits would have been the same. The reduced hemodilution with MiECC can also account for the better results obtained in this group in terms of lower O_2_ER_i_. The relationship between hyperlactatemia and hyperglycemia through the above mechanism was confirmed by Revelly et al. in 2005 [12] in an elegant study on cardiogenic or septic shock. The role of adrenergic agonists in this setting is well defined: in cardiogenic shock, these drugs are either endogenous or administered for cardiovascular therapy; in our model, they were endogenous in the majority of patients. None received epinephrine during CPB, and few received norepinephrine; however, unlike epinephrine, norepinephrine usually does not increase glucose production or induce an increase in plasma lactate concentration [13–15]. The two mechanisms leading to hyperlactatemia in various clinical conditions are therefore (1) anaerobic metabolism due to a poor DO_2_, and (2) excess lactate production due to glucose failing to enter the oxidative pathway and being degraded to lactate by the glycolytic pathway [13, 15, 16]. These mechanisms, if independently considered, lead to different acid–base balance conditions, the former being accompanied by metabolic acidosis and the latter not necessarily so. However, in the clinical conditions of this observational study, the acid–base balance is constantly maintained at a normal pH value by bicarbonate corrections applied by the perfusionist whenever the base excess starts decreasing. Therefore, we were unable to identify differences in hyperlactatemia related to different values of peak blood lactate. However, the evidence that only four patients showed hyperlactatemia without hyperglycemia and that only patients with a hyperlactatemia-hyperglycemia syndrome had significantly lower DO_2_ values seems to confirm that, in our specific clinical environment, hyperlactatemia and hyperglycemia are linked by the causative factor of a poor DO_2_. This leads on one hand to lactate production through the anaerobic pathway and on the other hand to a vicious cycle of lactate production due to the poor ability to use glucose through the aerobic pathway [3, 6, 11, 17]. Reduced oxygen content in cases of acute anemia is usually compensated by reduced blood viscosity with increased blood flow in the microcirculation and by a compensatory increase in cardiac output [18]. This last mechanism may be impaired during CPB, where pump flow is usually adjusted on the basis of the patient’s body surface area and temperature, not the Hb value. On the basis of our data, the main rationale for explaining hyperlactatemia during CPB is a DO_2_ inadequate to guarantee the needed oxygen consumption of the patient.

In the present study, we investigated the role of potentially modifiable factors related to CPB during CABG surgery in determining postoperative hyperlactatemia (e.g. due to inadequate perfusion) and hyperglycemia [19]. Our results demonstrate that a DO_2i_ < 270 mL/min/m^2^ with O_2_ER_i_ > 35% and low CI (< 2.4 L/min/m^2^) with SvO_2_ < 65% during CPB are associated with hyperlactatemia and hyperglycemia and DO_2i_ > 290 mL/min/m^2^ with O_2_ER_i_ < 25% and CI > 2.4 L/min/m^2^ with SvO_2_ > 75% during CPB are associated with a low incidence of hyperlactatemia and hyperglycemia. Various preoperative factors or comorbidities may create the right environment for hyperlactatemia during CPB. Age, female gender, congestive heart failure, low LVEF, hypertension, atherosclerosis, diabetes, preoperative Hb value, redo or complex surgery, and emergency procedures were found to be risk factors for hyperlactatemia by Demers and coworkers [20], who reported an hyperlactatemia incidence of 18%. Some of these factors were confirmed in our study, and other new factors were identified. However, our study population had a significantly shorter CPB duration and a lower degree of hemodilution during CPB. Given that both these factors seem to favor the onset of hyperlactatemia, the lower hyperlactatemia rate in our population is reasonably explained. The role of CPB duration in the development of hyperlactatemia during CPB has been highlighted in other studies [2, 20, 21]. Moreover, the additional volume of crystalloid in the cECC group resulted in significant hemodilution as indicated by the mean Hb values which were more than 2 g/dL greater for the MiECC group during CABG. This factor alone could have had a large impact on the other dependent variables, including lactate levels and oxygen delivery.

### Study limitations

Several limitations should be acknowledged. First, this is a single-centre study with a small sample size. Second, we did not know the microcirculation response for the higher Hb values in the MiECC group compared to the cECC group. Third, there were no inflammatory markers (cytokines) that could affect postoperative outcome, including DO_2i_. Fourth, eight pre- and intraoperative factors were found to be significantly associated with peak blood lactate levels during CPB at univariate analysis (i.e. age, isolated coronary operation, lowest pump flow/blood pressure, requirement of vasopressor or inotropic medications, lowest temperature, Hct, and DO_2i_) which were negatively correlated with peak blood lactate levels during CPB, whereas CPB duration and peak blood glucose were positively correlated with peak blood lactate levels during CPB. Notwithstanding this, the samples were homogeneous as for the characteristics. The Landing monitoring system was used for DO_2_ management during CPB; however, we did not record time durations < 280 mL/min/m^2^. Finally, the availability of perfusionists with and without skills for managing the MiECC technique is another limitation of the study.

## Conclusion

End-stage coronary artery disease patients undergoing myocardial revascularization with the MiECC technique had a higher DO_2i_ in relation to O_2_ER_i_ 20–25% compared to patients operated on with the cECC technique. MiECC patients showed a significant reduction in blood red cell units administered, in the incidence of peak intraoperative lactate, which correlated with reduced postoperative serum creatinine and shorter mechanical ventilation and ICU stay, as compared to cECC patients.

## Data Availability

The datasets used and analysed during the current study are available from the corresponding author on reasonable request.
